# An unexpected death after low anterior resection due to disseminated intravascular coagulation: A case report

**DOI:** 10.1016/j.ijscr.2020.05.045

**Published:** 2020-05-29

**Authors:** Jurij Ales Kosir, Mensur Salihovic, Primoz Sever, Jasna Klen

**Affiliations:** a Department of Abdominal Surgery, UMC Ljubljana, Zaloška Cesta 7, 1000, Ljubljana, Europe, Slovenia; b Department of Anesthesiology and Surgical Intensive Therapy, UMC Ljubljana, Zaloška Cesta 7, 1000, Ljubljana, Europe, Slovenia

**Keywords:** Disseminated intravascular coagulation, Rectal carcinoma, Postoperative death, Case report

## Abstract

**Introduction:**

Disseminated intravascular coagulation (DIC) is a blood clotting disorder that is characterized by intravascular thrombi formation and exhaustion of platelets and coagulation factors resulting in diffuse hemorrhage and multiple organ dysfunction.

**Presentation of case:**

We present a case report of a previously healthy middle-aged patient who was treated by low anterior resection for rectal carcinoma and suddenly went into unexpected circulatory shock on the 7th postoperative day. Despite our resuscitation efforts, he succumbed to the disease. The postmortem examination showed findings consistent with DIC.

**Discussion:**

The cause for DIC in this patient may be multifactorial. Sepsis, tumor-related factors and hereditary predisposition may have played a role. The role of blood components in treatment is not fully understood as they may worsen the inflammatory response.

**Conclusion:**

DIC can be the presenting sign of unrecognized sepsis and it can occur in all surgical patients. Even with prompt treatment, the disease can have a fatal course.

## Highlights

• Disseminated intravascular coagulation can develop due to unrecognized sepsis.
• It can have a life-threatening course even in young, previously healthy patients.
• Rotational thromboelastometry may aid in guiding the treatment.

## 1 Introduction

Disseminated intravascular coagulation (DIC) is a disorder of coagulation pathways that leads to intravascular activation of coagulation factors, fibrin deposition and thrombosis with organ dysfunction as a result of compromised perfusion [1]. The depletion of platelets and clotting factors leads to thrombocytopenia and coagulation factor deficiency resulting in bleeding diathesis [1].

DIC typically occurs as a secondary phenomenon and the inciting clinical events range from malignancies, blood transfusions, trauma, infections to perioperative complications [2]. In clinical practice, DIC can present anywhere along the spectrum from severe hemorrhage to multiple thromboses. Patients may experience bleeding from multiple sites and surgical patients may bleed into wounds [3]. Depending on the site of bleeding or thrombosis patients may develop dysfunction of the affected organs [4].

DIC is suggested by laboratory findings of consumption of coagulation factors together with increased fibrinolytic activity, resulting in thrombocytopenia, prolonged prothrombin time, activated partial thromboplastin time, and elevated levels of fibrin degradation products together with D-dimer [4]. Rotational thromboelastometry (ROTEM) may reveal prolonged clotting time and reduced clot strength accompanied to a hyperfibrinolytic state [5].

Therapy of DIC aims at treating the primary cause. Supportive therapy should be directed to correct tissue ischemia. Blood component therapy should be reserved for those who require a surgical procedure, are bleeding, or are at high risk for bleeding complications [6]. Consumed plasma coagulation factors can be supplemented with infusions of fresh frozen plasma or with the administration of prothrombin complex concentrate [6,7]. Bleeding associated with hyperfibrinolysis can also be treated with antifibrinolytics [8]. The administration of heparin can be used to inhibit the coagulation cascade and should be considered in DIC with the predominance of thrombosis [8].

This article presents a case report of a healthy patient with a fulminant course of DIC that developed unexpectedly on the 7th postoperative day without previous signs of complications.

This case report adheres to Surgical Case Report Guidelines (SCARE) [9].

## 2 Presentation of case

A 43-year old male patient without significant medical history was admitted to the surgery ward of a tertiary center for treatment of rectal cancer. There was no personal history of cardiovascular risk factors or a family history of premature atherosclerosis, thromboembolic disease or sudden death. Physical examination revealed a well-nourished man with normal cardiothoracic and abdominal examinations.

The tumor was obstructing the rectum and was proven endoscopically and with CT of the abdomen with an estimated stage of cT3 N0 M0. Due to the location of the tumor in the upper third of the rectum, the patient did not receive neoadjuvant therapy. CT also showed calcifications in both adrenal glands with no identifiable cause for this. The relevant results of the initial blood analysis are shown in Table 1. The liver function tests, international normalized ratio (INR) and creatinine were normal.

**Table 1:** Relevant laboratory results measured the day before the operation.

Hemoglobin	Platelets	CRP	CEA	CA 19-9
[13–17 g/dl]	[150–410 × 10^9^/l]	[<0.5 mg/l]	[<4,2 μg/l]	[<37 kU/l]
13,0 g/dl	422 × 10^9^/l	23 mg/l	22,6 μg/l	108 kU/l

CRP – C-reactive protein, CEA – carcinoembryonic antigen, CA19-9 – cancer antigen 19-9.

The patient was operated and a low anterior resection with colorectal anastomosis was performed. No distant spread of the disease was found during the operation. A drain was placed in the abdominal cavity and the bowel specimen was sent for histological evaluation, which confirmed a mucinous rectal adenocarcinoma. The pathologist reported the tumor stage to be pT4a N0 and confirmed R0 resection. For the first six days, his postoperative course was uneventful and the patient was receiving saline infusions until oral feeding commenced, low-molecular-weight heparin for antithrombotic prophylaxis and anti-pain medication. There were no significant leaks through the drain, which was removed on the 3rd postoperative day (POD).

On the 7th postoperative day, the patient suddenly went into circulatory shock. His blood pressure dropped down to 90/50 mm Hg, his heart rate was 130 beats per minute, respiratory rate was 32 breaths per minute and his temperature was 37.1 °C. His initial arterial oxygen saturation measured 78% with no added oxygen. Urgent contrast-enhanced CT of the thorax did not reveal signs of pulmonary embolism or fluid in the pericardial sac. The ECG did not show signs of cardiac ischemia either. Contrast-enhanced CT of the abdomen revealed a collection next to the colorectal anastomosis suggestive of an anastomotic leak. Blood cultures were taken and were positive in a few days for the growth of Escherichia coli, Streptococcus parasanguinis and Enterococcus faecium. Significant laboratory findings and the arterial blood gas analysis of that time are shown in Table 2, Table 3. The liver function tests, troponin I and creatinine were normal.

**Table 2:** Relevant laboratory results measured at the start of the patient's deterioration.

Hemoglobin	Platelets	CRP	INR	D-dimer
[13–17 g/dl]	[150–410 × 10^9^/l]	[<0.5 mg/l]	[<1,0]	[<500 μg/l]
10,8 g/dl	126 × 10^9^/l	184 mg/l	1,22	12864 μg/l

CRP – C-reactive protein, INR – international normalized ratio.

**Table 3:** Arterial blood gas analysis measured at the start of the patient's deterioration.

pH [7.35–7.45]	pCO_2_ [35–45 mmHg]	pO_2_ [75–100 mmHg]	HCO_3_^−^ [22–26 mmol/l]	Base deficit [-2-2 mmol/l]	Lactate [<2.0 mmol/l]
7,38	26 mmHg	74 mmHg	15 mmol/l	8,4 mmol/l	5,2 mmol/l

When inserting a central venous catheter, it was evident that blood clotting was disturbed as a large subcutaneous hematoma developed. During this time there was evident neurological dysfunction, manifesting itself as decreased levels of consciousness and confusion. Despite high-flow oxygen support and adding intravascular fluids his condition continued to worsen and he had to be intubated and started on high-dose vasopressive support.

The patient was urgently reoperated by a consultant abdominal surgeon. A swab of the abdominal cavity was taken for microbiological analysis and later results revealed the growth of Escherichia coli, Enterococcus faecalis and *Clostridium innocuum*. There was some blood concentrated in the pelvis. Minimal manipulation of the descending colon started diffuse bleeding in the pelvic region which was packed to control the bleeding. Leakage of the colorectal anastomosis was found and following the rules of damage control surgery the anastomosis was resected with a linear stapler. We did not perform an anastomosis or stoma.

**Fig. 1:**
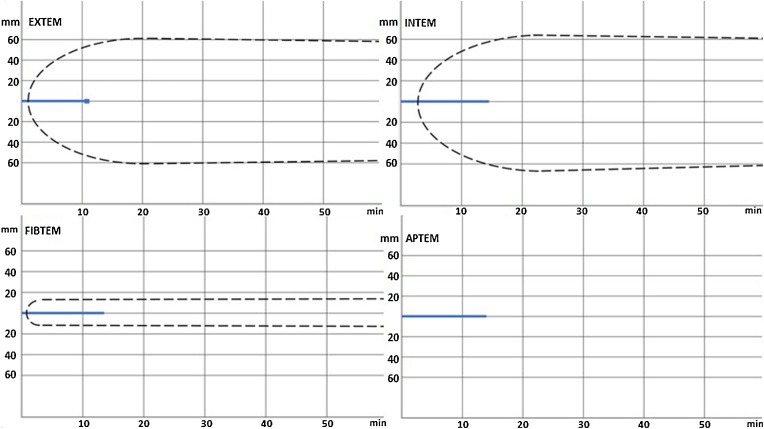
The initial rotational thromboelastometry revealed severely prolonged clotting times in all four modalities (clotting times measured by EXTEM, INTEM, FIBTEM and APTEM were 679 s, 879 s, 853 s and 807 s, respectively). Other parameters were unmeasurable due to the severity of the blood clotting disorder.

After closing the laparotomy, the patient sustained a cardiopulmonary arrest on the operating table from which he could not be resuscitated. At the beginning of the operation, ROTEM demonstrated a severe hypocoagulable state (Fig. 1). During the procedure and resuscitation efforts, the patient received a total of 8 units of packed red blood cells, 8 units of fresh frozen plasma, 2 packs of platelets, 4000 units of prothrombin complex concentrate and 8 g of fibrinogen. ROTEM did not improve after giving the blood components. The postmortem examination showed severe coagulopathy with multiple thrombi in the vessels consistent with DIC without any signs of comorbidities.

## 3 Discussion

DIC usually occurs in patients with underlying life-threatening diseases [1]. Only a few cases of DIC associated deaths in middle-aged surgical patients have been reported so far [[10], [11], [12], [13]].

The occurrence of DIC in our patient may have been triggered by the release of coagulant proteins from manipulation of the tumor and from an infection that was associated with the bowel anastomotic leak and was also later confirmed with positive blood cultures and positive growth from swabs of the abdominal cavity. The carcinoma in our patient had a mucinous component, which may have also played a part in initiating the thrombosis. Mucinous neoplasms are known to carry greater risk for thrombosis associated events due to the expression of procoagulant factors by tumor cells [1].

At first, we treated the presenting symptoms. The laboratory tests showed mildly increased INR. INR can be found to be within normal ranges in nearly one-half of patients with DIC because of the presence of activated clotting factors within the circulating system [4]. However, we noted an increase of INR from its baseline value, which was measured before the operation. ROTEM later revealed severely disturbed all pathways of blood clot formation which guided our therapeutic approach by administering blood-clotting agents. Interestingly, ROTEM did not show any signs of hypercoagulability in the patient.

Other causes for a circulatory shock in a young patient include pulmonary embolism, cardiac tamponade, which were both ruled out on the CT of the thorax. The patient did not have any chest pain and his ECG, as well as cardiac enzymes were unremarkable making acute coronary syndrome or stress-induced cardiomyopathy less likely. It is also not usual for a bleeding diathesis of sudden onset to occur in the above-mentioned diseases [8].

During the operation, there was evident bleeding from the pelvis which required transfusions of blood components. This may have also played a role in worsening DIC, as blood components can worsen the course of DIC just like they may cause DIC through a transfusion reaction [2]. Blood components, however, did not restore the coagulation and platelet functions as the bleeding continued. The administration of clotting factors is justified in patients who have serious bleeding, even though the level of evidence of these measures improving the outcome is low [3,4].

Great caution is required when using antifibrinolytic agents in patients with DIC as fibrin deposition is a feature of DIC and fibrinolysis is needed for its resolve [13]. Antifibrinolytic agents should be reserved only to selected patients in which hyperfibrinolysis dominates in the clinical picture [13]. Our patient had neurological dysfunction which could have been due to microthrombi in the cerebral circulation and as such he was not a good candidate for treatment with antifibrinolytic agents.

## 4 Conclusion

The clinical course of DIC ranges from asymptomatic to life-threatening. It is important to think of DIC when treating postoperative patients who develop widespread microthromboses and profuse uncontrollable hemorrhage even in young healthy patients. There is no doubt that the major determinant of survival is the ability to identify the underlying trigger and manage it successfully.

## Declaration of Competing Interest

No conflicts of interest for all the authors.

## Funding

There is no funding.

## Ethical approval

This is a case report – no ethical approval is needed to publish this.

## Consent

The consent from patient’s wife was obtained.

## Registration of research studies

This is not a study – it is a case report.

## Guarantor

The Guarantor is Jurij Ales Kosir.

## Provenance and peer review

Not commissioned, externally peer-reviewed.

## CRediT authorship contribution statement

**Jurij Ales Kosir:** Writing - original draft, Data curation, Investigation. **Mensur Salihovic:** Writing - review & editing. **Primoz Sever:** Writing - review & editing. **Jasna Klen:** Conceptualization, Supervision, Writing - review & editing, Visualization.

## Glossary

CA 19-9: Cancer antigen 19-9
CEA: Carcinoembryonic antigen
CRP: C-reactive protein
CT: Computed tomography
DIC: Disseminated intravascular coagulation
ECG: Electrocardiography
INR: International normalized ratio
LAR: Low anterior resection
ROTEM: Rotational thromboelastometry
